# Aberrant pattern of regional cerebral blood flow in mild cognitive impairment: A meta-analysis of arterial spin labeling magnetic resonance imaging

**DOI:** 10.3389/fnagi.2022.961344

**Published:** 2022-09-01

**Authors:** Tong Tang, Li Huang, Yusi Zhang, Zuanfang Li, Shengxiang Liang

**Affiliations:** ^1^National-Local Joint Engineering Research Center of Rehabilitation Medicine Technology, Fujian University of Traditional Chinese Medicine, Fuzhou, China; ^2^Rehabilitation Industry Institute, Fujian University of Traditional Chinese Medicine, Fuzhou, China; ^3^College of Rehabilitation Medicine, Fujian University of Traditional Chinese Medicine, Fuzhou, China; ^4^Innovation and Transformation Center, Fujian University of Traditional Chinese Medicine, Fuzhou, China; ^5^Traditional Chinese Medicine Rehabilitation Research Center of State Administration of Traditional Chinese Medicine, Fujian University of Traditional Chinese Medicine, Fuzhou, China

**Keywords:** mild cognitive impairment, cerebral blood flow, arterial spin labeling, meta-analysis, activation likelihood estimation

## Abstract

In mild cognitive impairment (MCI), cognitive decline is associated with abnormal changes of cerebral blood flow (CBF). Arterial spin labeling magnetic resonance imaging (ASL-MRI) is an effective method for assessing regional cerebral blood flow (rCBF). However, the CBF estimated *via* ASL-MRI in MCI often differs between studies, and the consistency of CBF changes in MCI is unclear. In this study, 13 ASL-MRI studies with 495 MCI patients and 441 health controls were screened out from PubMed, Embase, Cochrane, Web of Science, Wanfang, and CNKI. An activation likelihood estimation (ALE) meta-analysis was performed to explore the brain regions with abnormal CBF in MCI. It showed that the decreased CBF in MCI was identified in the precuneus, inferior parietal lobule (IPL), superior occipital gyrus (SOG), middle temporal gyrus (MTG), and middle occipital gyrus (MOG), while the increased CBF in MCI was identified in the lentiform nucleus (LN) compared with healthy controls. The study characterized the abnormal pattern of regional CBF in MCI, which would promote our knowledge of MCI and might be used as a biomarker in clinic.

**Systematic review registration:**

https://www.crd.york.ac.uk/prospero/display_record.php?RecordID=259633.

## Introduction

Mild cognitive impairment (MCI) refers to the symptomatic predementia phase of Alzheimer's disease (AD) that does not meet the diagnostic criteria for dementia (Langa and Levine, 2014). Its clinical manifestation is characterized by subjective or objective progressive memory loss (Albert et al., 2011; Petersen, 2011). Surveys have shown that MCI prevalence in adults above age of 65 years is as high as 20% (Livingston et al., 2017), and about 10–15% of these patients progress to dementia annually (Ganguli et al., 2010; Varatharajah et al., 2019). Once progressed into dementia, it not only causes irreversible cognitive impairment, but also brings serious social and economic burden (Müller et al., 2020). Multiple studies have investigated that subjects with reduced cognitive ability often have low cerebral blood flow [CBF; (de la Torre, 2012; Zhao et al., 2013; Leardini-Tristão et al., 2020; Weijs et al., 2022)]. The decreased CBF is a key process in the development of cognitive decline (Hanyu et al., 2010). The pathogenesis of MCI is still unclear, but studies have shown that MCI patients present with altered CBF (Quattrini et al., 2021; Zhang et al., 2021).

Arterial spin labeling (ASL) is an MRI technique that reflects tissue perfusion (Soldozy et al., 2019). Recently, it has been gradually used to study cerebral perfusion patterns in MCI patients, the relationship between regional cerebral blood flow (rCBF) and cognitive function in MCI patients, and to predict the progression of MCI disease (Duan et al., 2021; Soman et al., 2021; Marterstock et al., 2022). Compared with single photon emission computed tomography (SPECT) and positron emission tomography (PET), ASL has many advantages such as safe, non-invasive, non-radiation and simple operation (Soldozy et al., 2019; Schidlowski et al., 2020). Moreover, the accuracy of cerebral perfusion maps obtained by ASL is similar to that of SPECT, and which is more sensitive to the area of abnormal cerebral perfusion reduction (Takahashi et al., 2014; Haller et al., 2016). ASL can detect signs of neurodegeneration and directly reflect the neurological activity of the brain (Lou et al., 2017; Dolui et al., 2020), which is helpful for the prevention, diagnosis and detection of diseases in clinical practice.

An increasing number of studies apply ASL to examine perfusion in MCI (Xie et al., 2019; Camargo and Wang, 2021). In a resting-state condition, parietal (Johnson et al., 2005; Alexopoulos et al., 2012; Wierenga et al., 2012; Lou et al., 2016), hippocampal (Kim et al., 2013; Okonkwo et al., 2014; Duan et al., 2020; Camargo and Wang, 2021), and temporal lobes (Wierenga et al., 2012; Ding et al., 2014; Wang et al., 2020; Camargo and Wang, 2021) often showed an abnormal perfusion in patients with MCI as compared with healthy control (HC). However, results across the studies are inconsistent. Cingulate gyrus, precuneus, angular gyrus, and thalamus have also been reported to have abnormal blood perfusion (Alexopoulos et al., 2012; Wierenga et al., 2012; Xekardaki et al., 2015; Wu et al., 2019). Although most studies have shown that MCI patients have hypoperfusion brain regions, other studies have found that MCI patients have hyperperfusion, such as the frontal lobe, hippocampus, and cingulate gyrus (Wierenga et al., 2012; Kim et al., 2013; Ding et al., 2014; Wu et al., 2019; Duan et al., 2020). This difference may be related to the sample size of the included population, statistical analysis methods etc. Studying the alterations in brain rCBF may contribute to early diagnosis of MCI. Previous systematic review has mainly reported on cerebral perfusion under ASL imaging in AD patients (Ma et al., 2017), and no reports have been seen on MCI patients under ASL imaging. Therefore, there is an urgent need for meta-analysis to determine the location of altered cerebral perfusion in MCI patients.

Activation likelihood estimation (ALE) is a common technique for performing coordinate-based meta-analysis of brain imaging (Alain et al., 2018). The method converges different studies and simulates the likelihood of coordinate distribution according to the algorithm (Laird et al., 2005; Wager et al., 2007; Costafreda, 2009; Tanasescu et al., 2016). Through random effect analysis, ALE values aggregated from different literature were compared with ALE values obtained from the null distribution, and multiple comparisons were made, and significance tests also provide reliability of the results (Turkeltaub et al., 2002; Humphreys and Lambon, 2015). To identify a consistent pattern of cerebral perfusion changes in MCI patients, we applied an ALE meta-analysis of CBF in MCI patients to provide a basis for the evaluation and treatment with MCI.

## Materials and methods

The meta-analysis is conducted in strict accordance with the requirements of the Preferred Reporting Items for Systematic Reviews and Meta-analysis (PRISMA) statement (Moher et al., 2009) and was registered at International Prospective Register of Systematic Reviews (https://www.crd.york.ac.uk/PROSPERO/), (numbers CRD42021259633).

### Search strategy

Pubmed, Web of Science, Embase, Cocrane, CNKI, and WanFang database were searched for articles through April 03, 2022. Researchers searched for the keywords (“mild cognitive impairment” or “MCI”) and (“arterial spin labeling imaging” or “ASL”) combined. All searched articles were imported into the literature management software Endnote to eliminate duplicate records.

### Selection criteria

Studies meeting the following criteria were included: (1) original research article published in a peer-reviewed journal; (2) diagnosis of patients with MCI; (3) CBF differences between MCI and HC were measured using resting-state ASL imaging; (4) reported 3D coordinates in Montreal Neurological Institute (MNI) or Talairach space.

Exclusion criteria: (1) unavailability of full text or raw data; (2) animal trial studies; (3) articles without MNI or Talairach coordinates provided; (4) studies published in duplicate or similar data sources; (5) non-research articles such as conferences, reviews, letters and books.

### Data extraction

Information extraction for the final included study was completed by two researchers independently and checked by a third researcher. Baseline information, ASL characteristics and coordinates were extracted from these studies. Baseline information included first author name, publication year, sample size, mean age of sample, sex, Mini-Mental State Examination (MMSE) score, and other characteristics which are shown in Table 1.

**Table 1:** Characteristics of the ASL studies included in the meta-analysis.

**References**	**Sample (female)**		**Mean age (SD)**	**MMSE (SD)**	**Scanner strength**	**Imaging technique**	**Software**	**FWHM**	**Threshold**
Alexopoulos et al. (2012)	MCI	24 (8)	69.6 (8.2)	NA	3.0 T	PASL	SPM5	12 mm	0.001, uncorrected
	HC	24 (16)	67.1 (6.1)	NA					
Ding et al. (2014)	MCI	17 (11)	71.38 (7.61)	25.5 (2.2)	3.0 T	pCASL	SPM8	6 mm	0.05, corrected
	HC	21 (13)	69.64 (5.88)	29.4 (1.0)					
Duan et al. (2020)	MCI	50 (32)	84.5 (3.6)	NA	1.5 T	CASL	SPM8	6 mm	0.05, corrected
	HC	58 (31)	83.4 (3.7)	NA					
Johnson et al. (2005)	MCI	18 (9)	73.3 (8.6)	27.7 (NA)	1.5 T	PASL	SPM99	12 mm	0.001, corrected
	HC	23 (13)	72.9 (8.2)	29.4 (NA)					
Kim et al. (2013)	MCI	25 (13)	67.6 (7.4)	NA	3.0 T	PASL	SPM5	12 mm	0.005, uncorrected
	HC	25 (16)	68.4 (5.6)	NA					
Lv et al. (2015)	MCI	37 (21)	67 (9)	26.9 (1.7)	3.0 T	PASL	SPM8	8 mm	0.05, corrected
	HC	30 (11)	52 (8)	29 (1.0)					
Michels et al. (2016)	MCI	16 (4)	75.5 (8.0)	28.5 (1.2)	3.0 T	pCASL	SPM8	6 mm	0.05, corrected
	HC	27 (10)	71.8 (4.4)	29.6 (0.7)					
Okonkwo et al. (2014)	MCI	23 (7)	73.25 (6.95)	26.96 (2.01)	3.0 T	pCASL	SPM8	8 mm	0.005, corrected
	HC	24 (12)	75.07 (6.3)	29.04 (1.02)					
Shang et al. (2021)	MCI	44 (18)	68.95 (6.77)	24.95 (0.82)	3.0 T	pCASL	SPM12	8 mm	0.05, corrected
	HC	50 (25)	68.16 (4.07)	28.28 (1.15)					
Shokouhi et al. (2018)	MCI	185 (111)	64.4 (7.5)	NA	3.0 T	pCASL	SPM12	8 mm	0.005, corrected
	HC	80 (61)	63.1 (7.2)	NA					
Wang et al. (2020)	MCI	26 (8)	73.85 (7.4)	27.35 (1.55)	3.0 T	PASL	SPM8	NA	0.05, corrected
	HC	27 (8)	74.26 (6.4)	28.33 (1.33)					
Wierenga et al. (2012)	MCI	20 (10)	74.8 (11.4)	NA	3.0 T	PASL	AFNI,FSL	NA	0.05, corrected
	HC	40 (27)	73.5 (6.8)	NA					
Xu et al. (2007)	MCI	10 (5)	77 (4.47)	27.8 (1.5)	3.0 T	PASL	AFNI,FSL	10 mm	0.05, corrected
	HC	12 (5)	70 (3.9)	29.6 (0.79)					

ASL, arterial spin labeling; HC, healthy controls; SD, standard deviation; MCI, Mild cognitive impairment; MMSE, Mini-Mental State Examination; FWHM, full width at half maximum; NA, not available; PASL, pulsed arterial spin labeling; CASL, continuous arterial spin labeling; pCASL, pseudocontinuous arterial spin labeling; SPM, statistical parametric mapping.

### Study quality assessment

The quality of the included literatures was assessed using a 10-point checklist based on a previous neuroimaging meta-analysis (Wang et al., 2016). The total scale score of 10 points was divided into three sections. It focused on the included characteristics of participants, the methods of image acquisition and analysis, and the results of the articles (see Supplementary Table 1 for details). Two evaluators independently evaluated the quality of the included studies, and in case of disagreement, it is resolved through discussion or negotiation with a third party.

### Statistical analysis

The consistency of rCBF changes in MCI estimated by ASL was analyzed *via* a meta-analysis of ALE using the BrainMap GingerALE v3.0.2 (http://brainmap.org/). The voxel coordinates of each study report were regarded as probability distributions to create ALE distribution maps (Kollndorfer et al., 2013). The x, y, and z peak activation coordinates of all the clusters were included as the input for the meta-analysis. The ALE meta-analysis was estimated using a cluster-level inference threshold of *P* < 0.05 (family-wise error correction) with 5,000 permutations and *P* < 0.05 in MNI space.

## Results

### Search results and study characteristics

**Figure 1:**
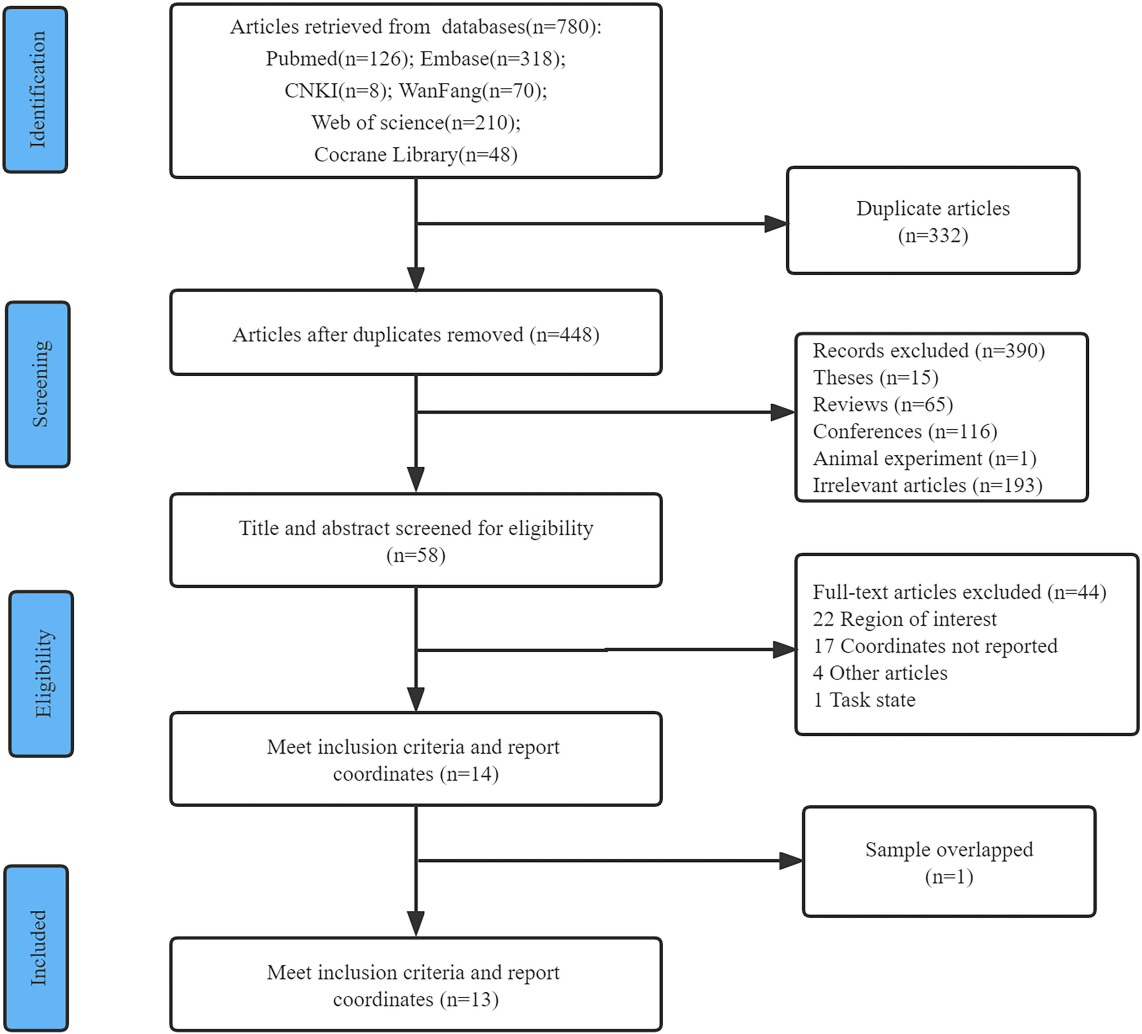
Flow diagram of the study selection procedure for the meta-analysis.

Seven hundred and eighty literature were obtained through initial retrieval, and 448 literature remained after removing duplicate literature. By reading the title and abstract, 390 articles were further excluded, including 15 theses, 65 reviews, 116 conferences articles, one animal experiment, and 193 other irrelevant studies. The full text was read according to the inclusion and exclusion criteria. Two articles described the same data set, one of which was excluded. Thirteen articles were finally included (Johnson et al., 2005; Xu et al., 2007; Alexopoulos et al., 2012; Wierenga et al., 2012; Kim et al., 2013; Ding et al., 2014; Okonkwo et al., 2014; Lv et al., 2015; Michels et al., 2016; Shokouhi et al., 2018; Duan et al., 2020; Wang et al., 2020; Shang et al., 2021), including 12 papers in English and one paper in Chinese, as shown in Figure 1 (literature screening flow chart).

**Figure 2:**
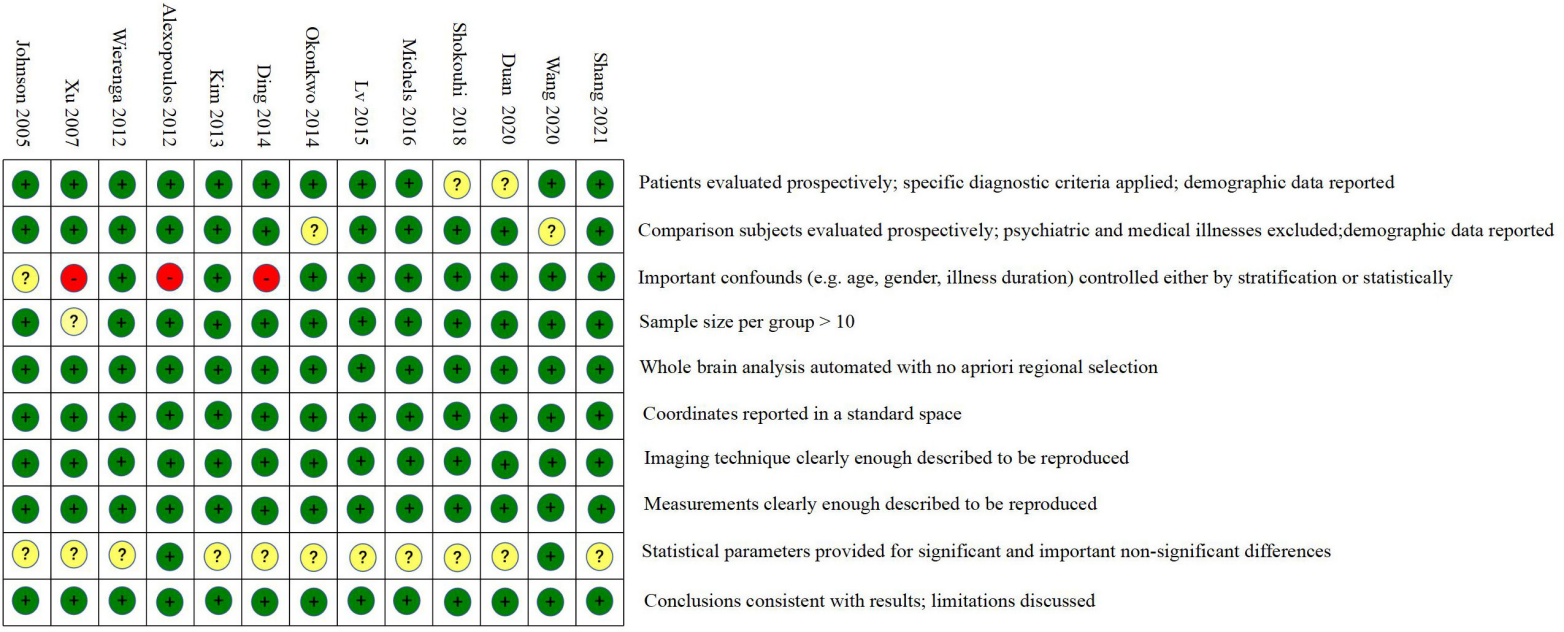
Literature quality assessment. The green circle means the information is clearly described in the study. The yellow circle means the information is partially met in the study. The red circle means the information is not described in the study.

This meta-analysis included a total of 936 patients, 432 males and 504 females, 495 MCI patients, and 441 HCs. The MCI group and the HC group in the 13 included studies were usually described by their characteristics, such as age, gender, MMSE, scanner strength, imaging technique, software, FWHM and threshold. Among these studies, 11 were conducted on the 3.0 T MRI scanning system, while the other two were performed on the 1.5 T MRI system. Regarding the techniques used to measure resting-state CBF in these studies, seven studies used pulsed ASL (PASL); five used pseudocontinuous ASL (pCASL), and the last one used continuous ASL (CASL). All included studies had an acceptable quality score of at least 8.0 (Figure 2, total score of 10). The main characteristics of these studies are shown in Table 1.

### Meta-analysis of studies of regional CBF differences

**Figure 3:**
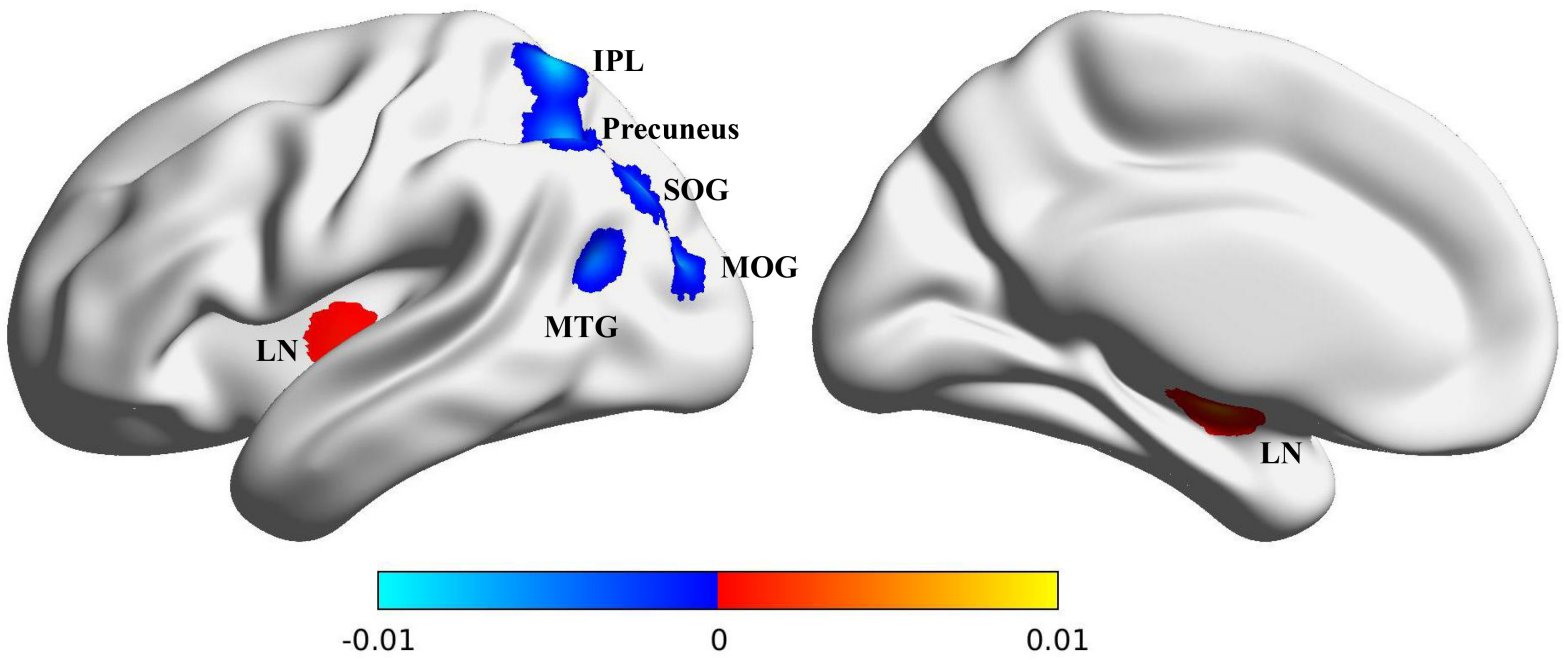
Brain map for the meta-analytic results of the ASL studies comparing rCBF differences between MCI patients and healthy controls. The gray region represents the outline of the brain. Significantly increased perfusion in the MCI is shown with warm color. Significantly decreased perfusion in the MCI is shown with cold color. The perfusion of precuneus, IPL, SOG, MTG and MOG is decreased, the LN is increased. ASL, arterial spin labeling; rCBF, regional cerebral blood flow; MCI, mild cognitive impairment. The color bar indicates the ALE value.

ALE meta-analysis was performed on CBF values of the 495 including MCI patients. Compared with HC, patients with MCI showed decreased rCBF in the precuneus, inferior parietal lobule (IPL), superior occipital gyrus (SOG), middle temporal gyrus (MTG), and middle occipital gyrus (MOG); patients with MCI showed increased regional CBF in the lentiform nucleus (LN), as shown in Figure 3 and Table 2.

**Table 2:** Clusters of regional CBF differences in patients with MCI compared to healthy controls.

	**Anatomical label**	**Peak MNI coordinate (x, y, z)**	**ALE-Z-value**	***p*-value (ALE)**
Decreased regional CBF	Precuneus (BAs 7 and 31)	−28, −70, 32	3.91	0.000046
	IPL (BAs 39 and 40)	−44, −60, 52	3.52	0.00022
	SOG (BA 19)	−36, −78, 38	3.47	0.00025
	MTG (BA 39)	−40, −64, 20	3.44	0.00029
	MOG (BA 19)	−30, −82, 18	3.38	0.00036
Increased regional CBF	LN (lateral globus pallidus and putamen)	−24, −10, −10	4.20	0.000014

CBF, cerebral blood flow; MCI, mild cognitive impairment; MNI, Montreal Neurological Institute; ALE, activation likelihood estimation; BA, brodmann area IPL, inferior parietal lobule; SOG, superior occipital gyrus; MTG, middle temporal gyrus; MOG, middle occipital gyrus; LN, lentiform nucleus.

## Discussion

In this study, a coordinate-based ALE meta-analysis was applied to investigate cerebral perfusion in MCI. 13 eligible studies with 495 MCI patients were analyzed. The CBF of the precuneus, IPL, SOG, MTG, and MOG was decreased, and the CBF of the LN was increased.

CBF is a critical biomarker of metabolic and functional activity in the brain (Zhang et al., 2017). The decrease in cerebral blood perfusion reflects the decrease of cerebral blood oxygen and energy metabolism, which is closely related to the changes in brain structure and function (Wang et al., 2013). Any sustained decrease in rCBF may affect tissue function and lead to local brain damage, which may affect cognition (Daulatzai, 2017). In this study, MCI showed hypoperfusion in the IPL, MTG, precuneus, SOG, and MOG. The IPL and MTG are areas where specific cognition is located (Cao et al., 2021) and are involved in attention and language processing (Caspers et al., 2013; Dong et al., 2021). The precuneus is associated with spatial memory (Deconinck et al., 2015) and extensively connects cortical and subcortical structures, which plays an important role in the default network (Koch et al., 2018; Chen et al., 2020a). It has been shown that amyloid deposition occurs in the precuneus, subparietal lobule, and temporal lobe in MCI patients (Trivedi et al., 2008; Huang et al., 2013; Rubinski et al., 2020). Moreover, rCBF in the precuneus, parietal and temporal lobes correlated with disease severity and memory performance as measured by the Clinical Dementia Rating Scale, which is consistent with the results of the present study (Wang et al., 2013). The SOG and MOG are associated with visual acuity and may be related to the process of consolidation of visual memory in cognition (Song et al., 2017; Sariah et al., 2020). The previous studies have found that the brain is modularized. It has been showed that the precuneus, SOG, and MOG, which are located in the occipital lobe, form a tightly connected module (Mastrandrea et al., 2017). The temporal lobe is involved in the other modules which are connected to the occipital cluster through the precuneus, which plays an important role in cognition processes (Dima et al., 2020). Several studies have found that these brain regions with reduced CBF may reflect distal functional deficits caused by structural neuronal damage. Therefore, the decreased rCBF in precuneus, IPL, SOG, MTG, and MOG observed in this study may be a reflection of pathophysiological processes, reflecting early vascular dysfunction and neuronal degeneration in MCI.

The LN is a region of the basal ganglia that is made up of the internal and external globus pallidus and the putamen (Herrero et al., 2002). The LN may be related to attention, working memory, reward and executive function in degenerative diseases (Li et al., 2021). In the present study, hyperperfusion was found in the LN. However, Ding et al. found hyperperfusion in the bilateral frontal lobes and right inferior temporal gyrus in MCI patients (Ding et al., 2014). In contrast, research has shown that hyperperfusion is present in the left hippocampus and right inferior temporal gyrus in aMCI patients compared with normal subjects (Dai et al., 2009). The presence of hyperperfusion in the LN in MCI patients may be related to compensatory mechanisms. The brain maintains higher neural activity by increasing blood oxygen and energy metabolism (Howarth, 2014; Wang et al., 2020). One study found that the mean CBF of the precuneus and postcentral gyrus in the aMCI group was positively correlated with cognitive level, but the result was not applicable in the control group (Wang et al., 2020), which laterally demonstrated the existence of compensatory mechanisms in MCI patients. The regions of compensation reported in the studies are different. Some studies have suggested that increased perfusion in the frontal lobe typically occurs during early cognitive decline (Park and Reuter-Lorenz, 2009; Clément and Belleville, 2010). With the gradual deterioration of cognition, the perfusion of frontal lobe is decompensated. In contrast, other compensatory pathways, such as the basal ganglia, cingulate gyrus, hippocampus, amygdala and other brain regions show increased perfusion (Alsop et al., 2008; Clément and Belleville, 2010; Chen et al., 2011; Ding et al., 2014). We speculate that beyond their association with the different degrees of cognitive impairment, the regions of compensation are also related to imaging technique, statistical analysis, and different reference standards.

In this study, the abnormal brain regions in MCI were consistent with other studies. In the meta-analysis regarding fluorodeoxyglucose PET (FDG-PET) in MCI patients, it was found that in the precuneus, MTG, and IPL glucose metabolism was reduced (He et al., 2015). In the brain atrophy study, structural atrophy was observed in the left MTG and right pallidum in MCI (Chen et al., 2020b). In addition, PET study showed decreased glucose metabolism in MCI such as cingulate gyrus, angular gyrus, and middle frontal gyrus (He et al., 2015). And brain atrophy was noted in the bilateral hippocampus, parahippocampal gyrus, amygdala and right insula (Chen et al., 2020b). These brain regions are associated with a wide range of cognitive functions such as attention, memory, and mood (Yamasaki et al., 2002; Sidhu et al., 2013; Li et al., 2017; Cao et al., 2018; Tibon et al., 2019). Our findings demonstrate that changes in brain activity in these brain regions may be an early imaging marker of MCI.

There are some limitations of this study. First, the number of included studies was relatively small. On the one hand, ASL research for patients with MCI is sparse. On the other hand, some studies did not report 3D coordinates due to different research methods, and raw data were difficult to obtain. Second, little research has differentiated MCI subtypes and this may have an impact on the results. Third, all the included articles were cross-sectional studies. The CBF changes as the MCI progresses are still unclear. Therefore, longitudinal comparison studies should be added in future.

In conclusion, the meta-analysis identified the abnormal region of CBF in MCI, which may contribute to the cognitive decline observed in patients with MCI. The alterations in rCBF may be used as an objective imaging marker for early diagnosis of MCI in the clinical.

## Data availability statement

The original contributions presented in the study are included in the article/Supplementary material, further inquiries can be directed to the corresponding author.

## Author contributions

SL, TT, and LH designed the whole study. TT, LH, YZ, and ZL searched and selected the studies, analyzed the data, prepared figures, and drafted the article. TT and SL undertook the statistical analysis. TT, LH, YZ, and SL participated in the interpretation of data. TT and LH wrote the manuscript. SL revised the manuscript. All authors read and approved the final manuscript.

## Funding

This work was supported by the grants from the Youth Science Foundation of Fujian Provincial Health Commission (2019-1-65), the National Natural Science Foundation of China (82004440), Natural Science Foundation of Fujian Province (2021J01961), and Scientific Research Foundation for the High-level Talents funded by Fujian University of Traditional Chinese Medicine (X2019002-talents).

## Conflict of interest

The authors declare that the research was conducted in the absence of any commercial or financial relationships that could be construed as a potential conflict of interest.

## Publisher's note

All claims expressed in this article are solely those of the authors and do not necessarily represent those of their affiliated organizations, or those of the publisher, the editors and the reviewers. Any product that may be evaluated in this article, or claim that may be made by its manufacturer, is not guaranteed or endorsed by the publisher.
