# Supplementary material for: Aberrant pattern of regional cerebral blood flow in mild cognitive impairment: A meta-analysis of arterial spin labeling magnetic resonance imaging
Source: Front Aging Neurosci. 2022 Sep 1;14:961344. doi: 10.3389/fnagi.2022.961344 (PMC9475306; doi:10.3389/fnagi.2022.961344)
Supplement: Supplementary file 1 [file Table_1.DOCX]

**Aberrant pattern of regional cerebral blood flow in Mild cognitive impairment: an meta-analysis of arterial spin labeling magnetic resonance imaging**

Tong Tang^1,2,3 †^, Li Huang^1,2,3 †^, Yusi Zhang^1,2,3^, Zuanfang Li^1,5^ Shengxiang Liang^1,2,4,^*

1. National-Local Joint Engineering Research Center of Rehabilitation Medicine Technology, Fujian University of Traditional Chinese Medicine, 350122 Fuzhou, Fujian, China

2. Rehabilitation Industry Institute, Fujian University of Traditional Chinese Medicine, 350122 Fuzhou, Fujian, China

3. College of Rehabilitation Medicine, Fujian University of Traditional Chinese Medicine, 350122 Fuzhou, Fujian, China

4. Traditional Chinese Medicine Rehabilitation Research Center of State Administration of Traditional Chinese Medicine, Fujian University of Traditional Chinese Medicine, 350122 Fuzhou, Fujian, China

5. Innovation and Transformation Center, Fujian University of Traditional Chinese Medicine, Fuzhou, Fujian, 350122,China

*Correspondence: sxliang@fjtcm.edu.cn (Shengxiang Liang)

^†^These authors contributed equally.

**Supplementary information**

**Table S1.** Quality assessment checklist

| **Category 1: Subjects** |
| --- |
| 1. Patients evaluated prospectively; specific diagnostic criteria applied; demographic data reported. |
| 2. Comparison subjects evaluated prospectively; psychiatric and medical illnesses excluded; demographic data reported. |
| 3. Important confounds (e.g. age, gender, illness duration) controlled either by stratification or statistically. |
| 4. Sample size per group > 10. |
| **Category 2: Methods for image acquisition and analysis** |
| 5. Whole brain analysis automated with no apriori regional selection. |
| 6. Coordinates reported in a standard space. |
| 7. Imaging technique clearly enough described to be reproduced. |
| 8. Measurements clearly enough described to be reproduced. |
| **Category 3: Results and Conclusions** |
| 9. Statistical parameters provided for significant and important non-significant differences. |
| 10. Conclusions consistent with results; limitations discussed. |

When criteria were partially met, 0.5 points were assigned. The aim of this rating was to describe the completeness of published studies with a numeric score in order to aid readers, and it is not intended to critique the investigators or the work itself.
